# Juvenile Justice, Technology and Family Separation: A Call to Prioritize Access to Family-Based Telehealth Treatment for Justice-Involved Adolescents' Mental Health and Well-Being

**DOI:** 10.3389/fdgth.2022.867366

**Published:** 2022-05-23

**Authors:** Marina Tolou-Shams, Eraka Bath, Jeanne McPhee, Johanna B. Folk, Michelle V. Porche, Lisa R. Fortuna

**Affiliations:** ^1^Department of Psychiatry and Behavioral Sciences, University of California, San Francisco, San Francisco, CA, United States; ^2^Department of Psychiatry, Jane and Terry Semel Institute for Neuroscience and Human Behavior, University of California, Los Angeles, Los Angeles, CA, United States; ^3^Department of Psychology, Drexel University, Philadelphia, PA, United States

**Keywords:** behavioral health equity, juvenile detention, family separation, telehealth, structural intervention

## Abstract

Separating children from families has deleterious effects on children's mental health and well-being, which is highly relevant for youth in juvenile detention and other out-of-home residential placements. Despite growth in the evidence of family-based interventions in mitigating adverse behavioral health outcomes for justice involved adolescents (JIA), gaps remain in intervention dissemination for JIA; this particularly true for those leveraging digital health technologies, a need that has intensified with the COVID-19 pandemic. Use of digital health technologies for JIAs is pressing to address structural barriers in maintaining JIA-family connections, but also to improve treatment access for detained JIAs. Court systems' capacity to support use of digital health tools, such as telehealth, appear promising. Data on the use of tele-conferencing in U.S. juvenile and family courts were collected from 456 juvenile justice professionals as part of a larger study on judicial decision making. Results suggest overwhelming adoption of video-conferencing for court hearings with only 40% of respondents reporting family court use prior to the onset of COVID-19, but majority (91%) now reporting its routine use. Youth participate from a range of settings, including detention, other residential placement, community-based behavioral health and in-home settings. The COVID-19 pandemic has created a shift in the uptake of video-conferencing platforms that could hold promise for future larger scale use across the juvenile justice system. Findings underscore feasibility and acceptability of technology requirements in key settings that should be leveraged for broad scale implementation of empirically supported family-based interventions to advance behavioral health equity for JIA.

## Introduction

The medical field is clear: Separating children from their families has deleterious effects on children's mental health and well-being (1). There has been a significant reduction in the number of children detained or incarcerated in the United States (U.S.) over the past decade; however, over 48,000 youth remain placed out-of-home in confined settings due to involvement in the juvenile legal or criminal justice system (2). For example, in California, on any given day, almost 5,500 youth (<21 years old) who come into contact with the juvenile justice system are separated from their families and housed in residential placement facilities (3).

## Family Contact During Youth Incarceration

Sustained contact and meaningful connection with family during incarceration is essential to preventing worsening mental health and associated outcomes, such as substance use and recidivism (4–6). Maintaining family ties during a youth's incarceration can include in-person or virtual visits, phone calls, and mail, each with its own barriers and benefits. Visitation policies (e.g., frequency, eligibility of youth, requirements of visitors) vary substantially by jurisdiction and families often face significant costs (transportation, childcare, time off work, etc.) to visit facilities in person. Phone call policies also vary by jurisdiction, with some providing brief (e.g., 10 min) calls free of charge and others not including any allotment of phone calls, increasing the burden on families, particularly those at longer distances. Limited research on family contact during youth incarceration suggests racial inequities, with White youth more likely to report more frequent and multiple forms of contact with family than Black youth (7).

The concomitant effect of family separation and the trauma and stress of incarceration for adolescents who enter the system already with significant unaddressed mental health needs is gravely concerning (8). Family-based interventions are the “gold standard” for improving behavioral health (i.e., mental health and/or substance use) outcomes and reducing recidivism among justice-involved adolescents (JIA) (9) but nationally in the U.S., youth and families are not typically receiving the opportunity to access this type of “gold-standard” intervention, prior to and during the COVID-19 pandemic. Specifically, structural barriers to family visitation, such as physical distance between the facility and the family's location, caregiver financial inability to travel, take time off work, and/or caregiving demands (e.g., care of other family members, such as siblings) have existed for decades. These are examples of types of structural determinants that create significant barriers, disrupt family connections, and have been documented to have significant long-term consequences for JIA and their families.

### Leveraging Technology to Promote Family-Based Treatment

Frameworks such as the User-Centered Design framework (10), which centers the needs and concerns of potential users during tool development and Participatory Informatics, which is derived from Community Based Participatory Research principles (11), prioritize the perspectives of those with lived experience in co-creating digital solutions. These models have been applied in various settings and can enhance the relevance and acceptability by ensuring the active involvement of target users in design phases. In line with these frameworks, Bath et al. (12) published a series of recommendations for how child and adolescent mental health professionals can and should play a pivotal role in the development and application of mobile health (mHealth) technologies to improve treatment access for JIA (12). Recommendations included, but were not limited to, the criticality of developing clinical system protocols that standardize the use of technologies for family-based interventions. Among these, the use of participatory informatics approaches to center youth and families in the development of such technologies and protocols was key to optimizing engagement. Another key recommendation was to augment workforce capacity and digital fluency by training clinicians and front-line juvenile justice system professionals in the use of mHealth technologies. Lastly, utilization of mHealth as a means to gather data to inform larger population treatment needs and reveal system level service gaps could have important policy-level and funding implications. Since those recommendations were published, the COVID-19 pandemic has intermittently halted in-person visitation in multiple juvenile detention facilities. The start of the pandemic required facilities to quickly pivot to implementing video-conferencing opportunities for brief family visitation (e.g., once per weekend) and/or attorney visitation. Yet, the use of these same video-conferencing platforms to conduct needed family-based behavioral health interventions (herein referred to as “family tele-behavioral health”) appears less typical.

Telehealth expansion has been documented to support improved access to necessary behavioral health care for minoritized youth and families (13). Nationwide, the U.S. disproportionately detains Black and Latinx adolescents who have been systematically disenfranchised from access to needed behavioral health care supports in the community that could have kept them out of the justice system (14). Using existing video-conferencing tools to deliver family tele-behavioral health interventions represents a critical and time-sensitive opportunity to address an overall dearth of services, particularly while JIA are detained and separated from their families. Ideally, these services would begin during time of detention and continue from detention to community reentry/at-home placement to support best outcomes. Many of these same families are disproportionately being impacted by the COVID-19 virus, which is resulting in dramatically higher rates of severe illness and mortality among ethnoracial minoritized communities in CA and throughout the U.S. (15). The confluence of adversities potentiated by the pandemic, both economic and health, increase the risk for trauma and chronic stress, particularly for those JIA who experience the negative impact of incarceration and separation from their families and communities.

### Key Structural Considerations for Implementation of Family-Based Intervention

Based on the above, it is imperative we capitalize on using technology for interventions and prioritize the development of family tele-behavioral health interventions, particularly when youth are forcibly separated from their families. This includes resolving the digital divide and addressing gaps in digital literacy and mitigating barriers unique to telehealth provision; for example, ensuring families have access to technology (e.g., access to laptops, tablets, phones with sufficient data plans, having Wi-Fi access, financial supports for technology access) and addressing linguistic barriers, accessibility considerations such as auditory and visual needs, as well as literacy concerns related to written telehealth platform directions and requirements for an email address. For example, in California, the Department of Juvenile Justice (CA DJJ) has family visitation information posted online; https://www.cdcr.ca.gov/juvenile-justice/djj-video-visiting-with-microsoft-teams/. Sites already conducting family tele-visitation should leverage this success and expand to deliver needed family tele-behavioral health therapeutic interventions. Additionally, providing technology support for families to increase their digital fluency and comfort in the navigation of online platforms is key. Systems responsible for JIA oversight, at state and local levels, must also engage in high-level realignment of facility structure and schedules to operationalize incorporation of mandated access to family-based telehealth intervention for JIA. Large-scale implementation of family tele-behavioral health intervention access requires both states and local jurisdictions (e.g., cities and counties) to partner with expert organizations (e.g., grassroot) and institutions (e.g., non-profit and academic), seasoned mental health clinicians (e.g., to train and deliver empirically-supported, family-based intervention via telehealth), digital health researchers (e.g., to study and track outcomes), policy-makers (e.g., for legislative advocacy and telehealth services reimbursement) and JIA and their families with lived experience. Justice-related stakeholders and systems are also critical to involve in this process of expanding access to family tele-behavioral health care while youth are detained. Judges, probation staff and attorneys for JIA are central players in identifying the behavioral health needs of detained youth and referring to (or in some cases mandating) mental health and/or substance use intervention. Studying the current use of video tele-conferencing in the juvenile justice system and for what purposes is a key first step in identifying how to leverage established video conferencing tools and procedures for the delivery of family tele-behavioral health services for separated JIA. We present recent data collected from a US national survey of juvenile and family courts to understand more about the current use of video teleconferencing in these settings and to inform next step considerations of ways to increase access to family tele-behavioral health services.

## Methods

### Procedures and Survey Content

Data on the use of tele-conferencing in juvenile and family court settings were collected from 456 juvenile justice professionals (i.e., judges, magistrates, juvenile court officers, or juvenile probation officers) as part of a larger parent study focused on judicial decision making. Staff were recruited from across the U.S. via professional listserv and department- and state-wide emails. Inclusion criteria for the parent study included currently holding a position as a judge, magistrate, juvenile court officer, or juvenile probation officer in the U.S. who has heard or worked with at least 20 juvenile delinquency cases in their tenure. Eligible participants completed an online Qualtrics survey at one time-point between December 3, 2020 and June 23, 2021. Survey questions asked professionals' demographic and jurisdictional information (e.g., location in the U.S.). Professionals were asked he use of tele-conferencing for court hearings in the family court settings prior to and during the COVID-19 pandemic. Those who endorsed family courts' tele-conferencing use were subsequently asked to identify from which locations youth and legal staff (e.g., judges, lawyers) joined tele-conferencing hearings. Descriptive analyses were conducted with this subset of survey items to understand the utilization rate and context of tele-conferencing in family court hearings. The study was approved by the institutional review board of Drexel University.

### Respondent Sample

**Figure 1:**
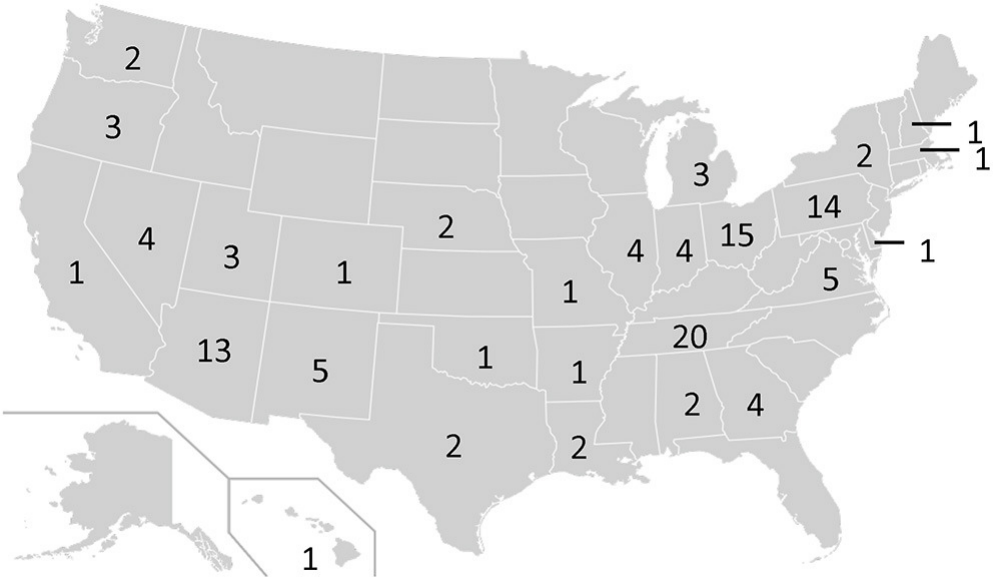
Geographic distribution of number of survey respondents from juvenile justice systems (*N* = 456; 15 participants did not note their geographic location).

Justice professional participants identified as male (50%), female (49%), or other/prefer not to say (1%). Participants predominantly identified as non-Hispanic (95%) and White (84%) with much less representation of Black (9%), American Indian/Native Alaskan/Native Hawaiian/Pacific Islander (3%) and Asian (1%) backgrounds. The age of participants ranged from 23 to 73 with an average age of 46 years, *SD* = 9.94. The majority of participants identified as probation officers (72%) while judges and magistrates represented 16% of the participants. Participants hailed from 28 distinct states (see Figure 1) and classified the jurisdictions in which they work as urban (38%), suburban (27%), or rural (35%).

## Results

**Figure 2:**
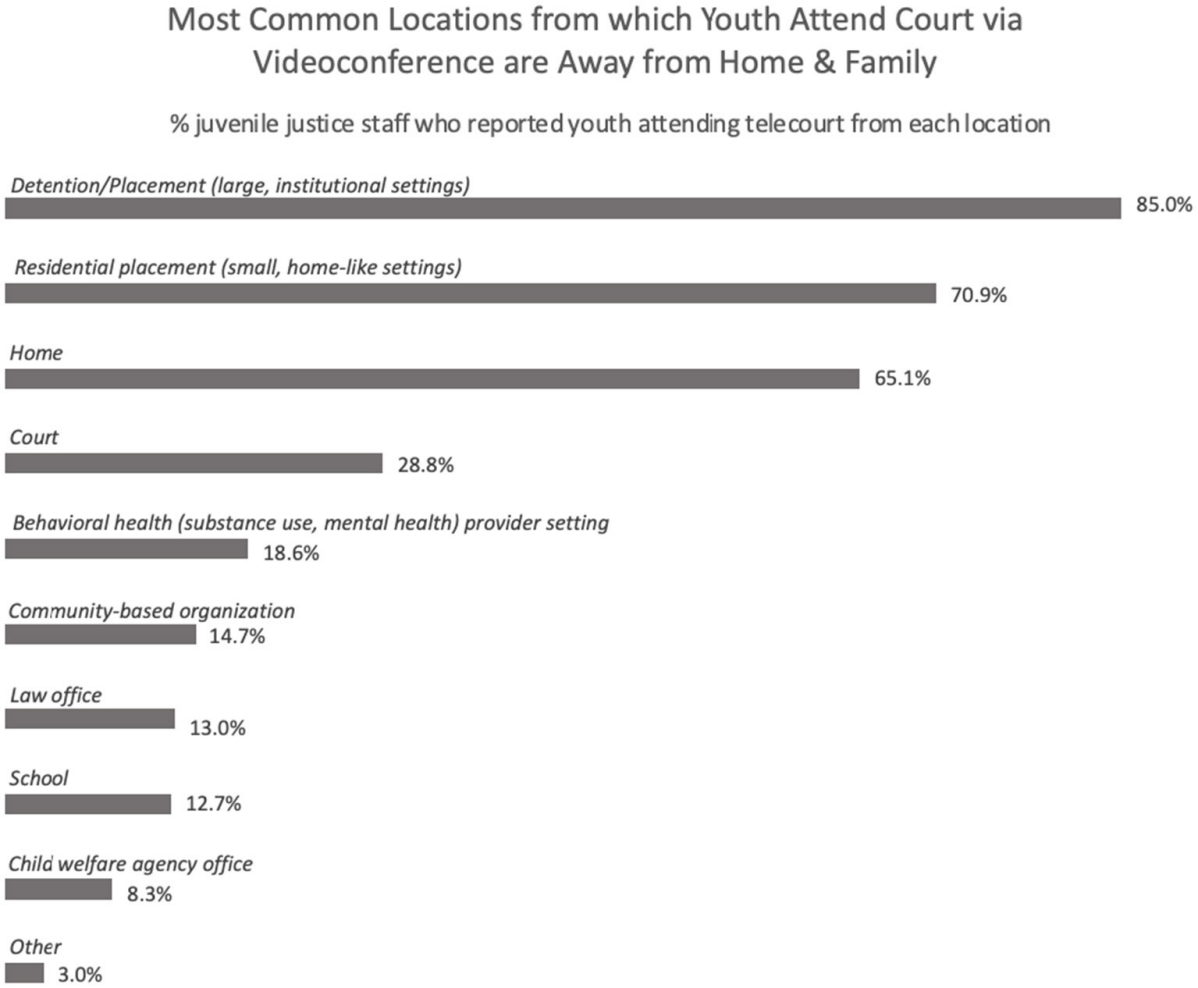
Locations from which youth attend court hearings using video tele-conferencing.

Data suggest that prior to the onset of the COVID-19 pandemic, only 40% of respondents reported family court use of tele-conferencing, but the majority (91%) now report its routine use. Respondents were asked where various parties, including youth, were located when using videoconferencing technology for family court hearings, with the option of endorsing more than one setting for each party (e.g., youth could be located in residential settings and while at a lawyer's office; see Figure 2). The majority (85%) of justice system staff identified that youth attend family court from detention. Most of the participants (71%) also indicated youth participate while in smaller, more home-like residential settings, followed by next largest proportion endorsing that youth participated in family court from home (65% of participants). Interestingly, one third of participants (33%) indicated that youth participated in family court hearings from either a behavioral health care setting or community-based organization at the time of the remote family court hearing suggesting that these systems also have some capacity to support video tele-conferencing.

## Discussion

In a time of such disaster and disparity, the COVID-19 pandemic has underscored the importance of rising to the challenge to leverage video conferencing and digital technology therapeutically for JIA and their families. Survey data collected from a national sample of justice professionals across 28 states suggest significant increased use of video tele-conferencing as a successful tool for court hearings, whether the youth is in detention or home. Data also suggest approximately one-third of youth are participating in these remote court hearings from behavioral health or community-based service settings. Accordingly, it appears the basic technology logistics associated with sustained uptake of video tele-conferencing between court and juvenile detention facilities have largely been figured out; video tele-conferencing tools are available to the courts and out-of-home settings, such as juvenile detention and small, home-like residential placements, which was not true prior to 2020 and the COVID-19 pandemic. Thus, the opportunity to leverage existing technology use to expand therapeutic interventions, specifically tele-behavioral health intervention access while youth are separated from their families in detention, is promising. Next steps include determining if there are differences in what is being offered via telehealth between smaller residential homelike settings and standard detention facilities and determining specific barriers to utilization of video conferencing tools to expand access to evidence-based family interventions (and other associated family-based care navigation and supports). Our data also suggest that video tele-conferencing for court hearings is commonly occurring from within the home while the JIA is living with family. This bodes well for the opportunity to expand access to continuity of care for family tele-behavioral health intervention when the JIA has re-entered the community from detention. It also provides promise for much needed family tele-behavioral health interventions for JIA who have never been detained but need behavioral health intervention while monitored in the community (e.g., through diversion programs).

The use of video teleconferencing to provide visitation has been highlighted as an opportunity to not only close the gaps and distance between those experiencing incarceration and their loved ones, but also as a supplement to in person visits. In the adult literature, video visitation has been highlighted as a way to mitigate transportation barriers, reduce behavioral infractions, and decrease risk of recidivism post release (16). Data on the implementation of tele-behavioral health services for adults in correctional facilities is increasingly showing promise, particularly in rural areas (17). Data on detained youth are more limited, suggesting adequate acceptability and no negative outcomes associated with telehealth care (18). Future research should focus on identifying what proportion of juvenile detention facilities are using these tele-conferencing tools to deliver family tele-behavioral health services vs. family tele-visitation vs. no family-based use of video tele-conferencing. Studies should also seek to understand at the local, state and national level what the barriers to delivering such family-based therapeutic care may be (18). For example, even if the logistics and availability of video tele-conferencing and other digital health tools no longer serve as barriers, addressing workface capacity and availability of clinicians trained in evidence-based family therapeutic interventions that are tailored to the multifaceted and unique needs of JIA youth and families is key. Ethical and system-related concerns around HIPAA and other required legal protections (e.g., special protections around confidentiality) associated with providing behavioral healthcare via telehealth to detained populations that are different than standard telehealth care should be explored and addressed. Determining whether such concerns and complexities serve as a barrier to extending existing video tele-conferencing capability to provide family tele-behavioral health services will be critical to understand for successful implementation.

Our current study data are not without limitations. First, this was not a comprehensive look across all states to identify accessibility of video tele-conferencing for court hearings for youth in detention, thus generalizability may be limited and access may be specific to only certain geographical locations. In addition, this was a self-selected sample of respondents, thus professionals from states or jurisdictions who may not be using these tools for court hearings may have been more likely to decline to participate overall. We did not include a survey item that asked about prior experience in using these types of technology tools to ascertain whether those who were responding were just more comfortable or familiar with the use of technology. Queries on acceptability and ease of use were also limited, so we were unable to ascertain nuances regarding ease of uptake and an understanding of the day-to-day challenges in utilization. Lastly, survey questions did not include items that asked justice professionals about their perspective on the use of existing video-conferencing tools for anything outside of court hearings (e.g., family visitation, family-based behavioral health services), which is an area for future research.

### Conclusions and Recommendations

Despite the aforementioned limitations, these data provide a first-time empirical snapshot from justice professional stakeholder perspectives of the use of video-conferencing tools within the juvenile court and detention settings. These data can help the field begin to consider next steps and recommendations related to how to use these digital tools and within these particular settings to advance health equity for youth who are forced to be separated from their families due to detention and out-of-home placement. Our recommendations include a need to: (1) urgently expand family tele-visitation services to also allow for family tele-behavioral health services; (2) leverage the policies and practices that are being used successfully for tele-court hearings for tele-behavioral health interventions to promote best outcomes for youth, including upon community re-entry. Interventions that start while in detention and continue during community re-entry give opportunity for a necessary continuum of care that builds trust, enhances engagement, and promotes best youth outcomes (19, 20). Care delivered via secure video-conferencing platforms (i.e., telehealth) provides a unique opportunity to continue with the same provider “from the inside to the outside,” and the field should be developing and testing outcomes associated with such interventions; (3) develop state-wide strategic plans with clear structural, fiscal and legislative aims to address juvenile justice behavioral telehealth infrastructure and implementation, and (4) make capital investments in aging infrastructure, justice staff professional development opportunities, and capacity building for community behavioral health providers to facilitate family tele-behavioral health service capacity and expansion for youth in detention.

## Data Availability Statement

The original contributions presented in the study are included in the article/supplementary material, further inquiries can be directed to the corresponding author/s.

## Ethics Statement

The studies involving human participants were reviewed and approved by Drexel University Institutional Review Board. The patients/participants provided their written informed consent to participate in this study.

## Author Contributions

MT-S conceptualized the manuscript topic and focus. EB contributed to the conceptualization and theoretical framework. JM provided empirical survey data, analysis, and interpretation. All authors participated in writing and editing process.

## Funding

This work was supported by the National Institute on Drug Abuse (MT-S K24DA046569; PI MT-S; K23DA050798; PI JF) and the National Institute on Mental Health (R34MH119433; PI MT-S). MP and MT-S also received support from the California Bench To School Initiative for this work.

## Conflict of Interest

The authors declare that the research was conducted in the absence of any commercial or financial relationships that could be construed as a potential conflict of interest.

## Publisher's Note

All claims expressed in this article are solely those of the authors and do not necessarily represent those of their affiliated organizations, or those of the publisher, the editors and the reviewers. Any product that may be evaluated in this article, or claim that may be made by its manufacturer, is not guaranteed or endorsed by the publisher.
